# Pancreatitis with a Normal Serum Lipase, a Rare Post-esophagogastroduodenoscopy Complication: A Case Report

**DOI:** 10.5811/cpcem.2022.3.55706

**Published:** 2022-07-27

**Authors:** Molly Sturlis, Karen McGrane

**Affiliations:** * Midwestern University Arizona College of Osteopathic Medicine, Glendale, AZ; † Madigan Army Medical Center, Department of Emergency Medicine, Tacoma, WA

**Keywords:** case report, pancreatitis, esophagogastroduodenoscopy, lipase

## Abstract

**Introduction:**

Pancreatitis after esophagogastroduodenoscopy (EGD) is not a common occurrence, particularly in the setting of a normal serum lipase. The lack of commonality may delay diagnosis and treatment in some patients presenting to the emergency department (ED) with abdominal pain after an otherwise uncomplicated procedure.

**Case Report:**

A patient with a history of gastroesophageal reflux disease presented to the ED with a complaint of abdominal pain and fever three days after an uncomplicated EGD. The patient was ultimately diagnosed with pancreatitis after a computed tomography showed pancreatic head inflammation, despite having a normal serum lipase. There were no other identified risk factors for pancreatitis in this case.

**Conclusion:**

This case serves to bring awareness to this potential procedural complication and the possibility of pancreatitis with a normal serum lipase.

## INTRODUCTION

Upper gastrointestinal endoscopy, also known as esophagogastroduodenoscopy (EGD), is a well tolerated, common diagnostic and therapeutic procedure with typical complications related to sedation, bleeding, perfor-ation, and infection.^1^ There have been fewer than an estimated 10 case reports of pancreatitis as a complication of post-upper endoscopies that do not involve ampullary cannulation; therefore, it is not routinely considered.^2^–^4^ We present a case of acute pancreatitis diagnosed in the emergency department (ED) three days after uncomplicated EGD. This case presented with an atypical location of pain, normal lipase level, and a lack of common risk factors.

## CASE REPORT

A 41-year-old White patient with a history of hypertension and gastroesophageal reflux disease (GERD) presented to the ED with fever and right mid to low abdominal pain after undergoing an uncomplicated esophagogastroduodenoscopy (EGD), with biopsies taken from the lesser curvature of the stomach, three days prior as part of the workup for persistent GERD. The pain began the night of the endoscopy and was originally in the mid to periumbilical region. The symptoms spontaneously improved the next day before progressively worsening, with the pain migrating to the right lower quadrant and reported fever of 38.9°C. The patient noted some nausea over the preceding days but had not vomited and had a normal appetite. The patient denied having diarrhea and stated they had only one bowel movement since the endoscopy, which was abnormally pellet-like, and they had not had a bowel movement or flatus in the prior 24 hours. The patient denied any previous abdominal surgeries. Current medications were bupropion, lisinopril, and omeprazole.

On physical examination, the patient was well appearing and afebrile, hypertensive to 147/102 millimeters of mercury, normal heart rate and respiratory rate, and in no acute respiratory distress. The abdomen was soft and non-distended with hypoactive bowel sounds. There was mild tenderness and voluntary guarding in the right lower quadrant, and the area of maximal tenderness was at McBurney’s point. No rebound tenderness, Rovsing’s, or Murphy’s signs were present.

**Image 1:**
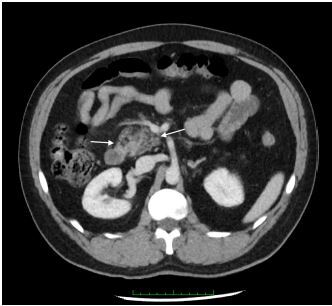
Axial computed tomography view showing inflammatory stranding of the pancreatic head (arrows).

**Image 2:**
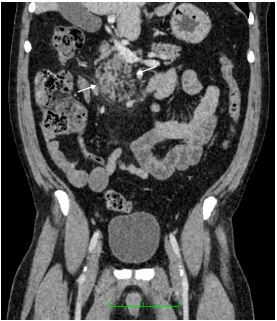
Coronal computed tomography view showing inflammatory stranding of the pancreatic head (arrows).

Laboratory findings were notable for a white blood cell count of 11.5 × 10^3^ per microliter (10^3^/μL) (reference range 4.5 – 13.0 10^3^/μL) with 74.1% neutrophils (reference range 38.5 – 76.5%), with the remainder of the complete blood count and complete metabolic panel within normal limits. Lipase level was also within normal limits at 36 units per liter (U/L) (reference range 13–60 U/L). A computed tomography of the abdomen and pelvis with intravenous contrast was obtained and indicated slight heterogeneity and enlargement of the pancreatic head with adjacent inflammatory fat stranding indicative of acute pancreatitis (Images 1 and 2).

There was no evidence of appendicitis. On re-evaluation, the patient stated that their pain had mildly improved after receiving ketorolac 15 milligrams intravenously and a normal saline fluid bolus and that the pain was now located periumbilically, similarly to how it felt at initial onset. The patient no longer had tenderness in the right lower quadrant, and there was no guarding or rebound tenderness.

**CPC-EM Capsule**

What do we already know about this clinical entity?

*While there have been some case reports of pancreatitis following uncomplicated esophagogastroduodenoscopy, it remains an uncommon complication*.

What makes this presentation of disease reportable?

*This case report features two uncommon occurrences - pancreatitis following uncomplicated esophagogastroduodenoscopy* (*EGD) in the setting of a normal lipase level. The two findings in conjunction make this an exceedingly rare event*.

What is the major learning point?

*Index of suspicion for pancreatitis should remain elevated in the setting of normal lipase enzyme levels. Consider pancreatitis in patients presenting with abdominal pain following EGD*.

How might this improve emergency medicine practice?

*This case identifies a rare cause of pancreatitis and may guide the clinician’s decision making process when seeing patients presenting with concerns following EGD*.

A right upper quadrant ultrasound was obtained to assess for gallstones as a possible cause of acute pancreatitis in this patient. It indicated no cholelithiasis with poor visualization of the pancreas due to bowel gas. The patient denied alcohol use. Prior record review indicated the patient had a history of borderline hypertriglyceridemia, with a level of 204 milligrams per deciliter (mg/dL) (reference range 0 mg/dL to 200 mg/dL) just three months prior to this presentation.

Given the improvement of symptoms and mild disease course, the patient was discharged home in stable condition and given strict return precautions as well as instruction to follow up with the gastroenterologist. On chart review, telephonic follow-up conducted by the patient’s gastroenterologist revealed improved symptoms, and biopsies from the EGD were normal, with no additional potential etiologies discovered for the clinical presentation and radiographic findings consistent with pancreatitis.

## DISCUSSION

In the absence of other plausible etiologies of acute pancreatitis, including alcoholism, cholelithiasis, hypertriglyceridemia, endoscopic retrograde cholangiopancreatography (ERCP), or offending medications, it is reasonable to explore a correlation between the recent upper endoscopy and findings consistent with pancreatitis on imaging.^5^ Pancreatitis is a well documented complication of ERCP, which involves ampullary cannulation but is not considered to be a complication of EGD.^6^ There have been few case reports of acute pancreatitis following EGD described in the literature. The mechanism by which uncomplicated EGD may cause acute pancreatitis is not well understood; it is theorized to be due to mechanical trauma or over insufflation during the procedure that causes local inflammation and ultimately may lead to development of acute pancreatitis within several days of the procedure.

This patient initially experienced periumbilical pain that migrated to the right lower quadrant, along with fever and some associated nausea, most suggestive of acute appendicitis. However, the CT did not support this diagnosis but instead indicated an acute pancreatitis. After pain control with ketorolac, the patient’s pain did again localize to the periumbilical region. The non-classic location of pain, the normal lipase level, and the lack of common risk factors for pancreatitis make its diagnosis a peculiar one; however, according to the guidelines put forth by American College of Gastroenterology, the diagnosis of acute pancreatitis should still have been made in this case. The guidelines suggest that to make the diagnosis, two of the three following criteria must be met: 1) abdominal pain; 2) serum amylase and/or lipase three times greater than the upper limit; and 3) characteristic findings on abdominal imaging.^7^

While this patient had normal lipase levels, they did present with abdominal pain and had a CT indicative of acute pancreatitis. The sensitivity of lipase in acute pancreatitis is reported as 64–100%, with a higher likelihood of a normal lipase either early in the presentation or later in the clinical course. Studies have shown the lipase levels rise within three to six hours of onset of pancreatitis and peak within 24 hours. Lipase levels may be persistently elevated for up to two weeks following acute pancreatitis.^8^ This patient’s symptoms began more than 24 hours prior to presentation, making a pre-elevation sample unlikely. There have been case reports in the past supporting the possibility of normal lipase levels, particularly in cases of acute on chronic pancreatitis, hypertriglyceridemia induced or in late presentations, none of which fit this clinical scenario.^9^–^13^

## CONCLUSION

Making the association between EGD and acute pancreatitis is important, since as it stands, pancreatitis is not considered a post-EGD complication. This fact may delay diagnosis and treatment in some patients presenting to the ED with abdominal pain after an otherwise uncomplicated procedure. This case report serves to bring awareness to this potential procedural complication.

## References

[1] Cohen J, Greenwald DA (2020). Overview of upper gastrointestinal endoscopy (esophagogastroduodenoscopy).

[2] Fadaee N, De Clercq S (2019). Gastroscopy-induced pancreatitis: a rare cause of post-procedure abdominal pain. J Med Cases.

[3] Ahmad Y, Anwar K, Dassum SR (2018). Acute pancreatitis after a routine esophagogastroduodenoscopy (EGD). Chest.

[4] Nwafo NA (2017). Acute pancreatitis following oesophagogastroduodenoscopy. BMJ Case Rep.

[5] Nevins AB, Keeffe EB (2002). Acute pancreatitis after gastrointestinal endoscopy. J Clin Gastroenterol.

[6] Vege SS (2021). Etiology of acute pancreatitis.

[7] Tringali A, Loperfido S, Costamagna G (2021). Post-endoscopic retrograde cholangiopancreatography (ERCP) pancreatitis.

[8] Ismail O, Bhayana V (2017). Lipase or amylase for the diagnosis of acute pancreatitis?. Clin Biochem.

[9] Yadav D, Agarwal N, Pitchumoni CS (2002). A critical evaluation of laboratory tests in acute pancreatitis. Am J Gastroenterol.

[10] Wang YY, Qian ZY, Jin WW (2019). Acute pancreatitis with abdominal bloating and distension, normal lipase and amylase. Medicine (Baltimore).

[11] Shafqet MA, Brown TV, Sharma R (2015). Normal lipase drug-induced pancreatitis: A novel finding. Am J Emerg Med.

[12] Agrawal A, Parikh M, Thella K (2016). Acute pancreatitis with normal lipase and amylase: an ED dilemma. Am J Emerg Med.

[13] Limon O, Sahin E, Kantar F (2016). A rare entity in ED: normal lipase level in acute pancreatitis. Turkish J Emerg Med.

